# Prognostic value of polarized macrophages in patients with hepatocellular carcinoma after curative resection

**DOI:** 10.1111/jcmm.12787

**Published:** 2016-02-04

**Authors:** Qing‐Hua Shu, Yong‐Sheng Ge, Hua‐Xing Ma, Xiao‐Qiang Gao, Jing‐Jing Pan, Dong Liu, Ge‐Liang Xu, Jin‐Liang Ma, Wei‐Dong Jia

**Affiliations:** ^1^ Anhui Province Key Laboratory of Hepatopancreatobiliary Surgery and Department of Hepatic SurgeryAnhui Provincial HospitalAnhui Medical UniversityHefeiChina; ^2^ Department of Thyroid Breast SurgeryPuai HospitalTongji Medical CollegeHuazhong University of Science and TechnologyWuhanChina; ^3^ Department of General SurgeryLu'an People's HospitalLu'anAnhui ProvinceChina

**Keywords:** CD11c, CD206, hepatocellular carcinoma, polarization, tumour‐associated macrophages

## Abstract

As the most predominant tumour‐infiltrating immune cells, tumour‐associated macrophages (TAMs) are significant for fostering tumour growth, progression and metastasis. CD68‐positive TAMs display dissimilarly polarized programmes comprising CD11c‐positive pro‐inflammatory macrophages (M1) and CD206‐positive immunosuppressive macrophages (M2). The aim of this study is to determine the prognostic significance of diametrically polarized TAMs in hepatocellular carcinoma (HCC) and their application to risk stratification of patients according to their specific prognostic values. This study included 80 consecutive patients with HCC, and we evaluated diametrically polarized functional status of macrophages by immunohistochemical staining of CD68, CD11c and CD206. Prognostic values and clinicopathologic features were assessed in these patients. High *versus* low CD11c‐positive TAM density (*P* = 0.005) and low *versus* high CD206‐positive TAM density (*P* = 0.002) were associated with better overall survival, whereas CD68‐positive TAM density had no prognostic significance (low *versus* high, *P* = 0.065). Furthermore, the presence of these positive staining macrophages did not show any prognostic significance for recurrence‐free survival (all *P* > 0.05). Multivariate Cox regression analysis identified CD11c‐positive and CD206‐positive TAMs as an independent prognostic factor (*P* < 0.001, *P* = 0.031, respectively). Intratumoural infiltration of diametrically polarized TAMs, a novel identified independent prognostic factor for survival in patients with HCC, could be combined with the TNM stage and the Barcelona Clinic Liver Cancer stage to improve a risk stratification system.

## Introduction

Hepatocellular carcinoma (HCC) represents the third leading cause of death from cancer worldwide and the fifth most common type of malignant tumour worldwide 1. Although there are improvements in the diagnosis and treatment of HCC, results are impaired by a high recurrence rates for 5 years (50–70%) and tumour‐related death (30–50%) 2, 3, 4. Traditionally, in clinical settings, the prediction of HCC outcome is based on one of the tumour staging systems [*i.e*. Barcelona Clinic Liver Cancer (BCLC), TNM, Japan Integrated Staging and cancer of the liver Italian programme] 3, 5. These different staging systems are mainly based on the tumour size, number of nodules, tumour invasion depth, lymph node metastasis, distant metastasis and severity of the liver disease. However, ignoring the role of the tumour microenvironment, these clinicopathological factors cannot provide complete prognosis assessment. Partial patients with advanced‐stage cancer can remain stable for years, whereas some early‐stage patients progress rapidly 6. Therefore, identification of specific biomarkers or stratification systems that can be used for more accurate prognostic prediction in patient survival is required immediately.

Chronic inflammatory conditions increase the risk of different forms of cancer. Patients with chronic hepatitis have an increased risk of developing liver cancer 7. Inflammation has a key role in the tumour microenvironment and has recently been recognized as a novel hallmark of cancer because of its association with the pathogenesis in many types of cancer 8, 9, 10. Macrophages are deemed to have a critical role in orchestrating the cancer‐associated inflammation. Macrophages are recruited by chemokines such as chemokine C–C motif ligand 2 and macrophage colony‐stimulating factor (M‐CSF) and are produced mainly by tumour cells 11. Macrophages respond to this microenvironment and have commonly been divided into two classes: ‘classically activated, pro‐inflammatory' M1‐polarized phenotype and ‘alternatively activated, immunosuppressive' M2‐polarized macrophages 12. Pro‐inflammatory classically activated by interferon‐gamma(IFN‐γ) or lipopolysaccharide (LPS), known as M1. The M1‐polarized macrophages secrete tumour necrosis factor α (TNF‐α), interleukin (IL)‐6, and monocyte chemotactic protein‐1 13, 14. Immunosuppressive alternatively activated by interleukin (IL)‐13 or IL‐4, known as M2. The M2‐polarized macrophages secrete IL‐10, transforming growth factor‐β, and alternative macrophage activation‐associated CC chemokine‐1, and promoting angiogenesis, tissue remodelling, and repair 13, 14. Macrophages that infiltrate tumour tissues, also termed tumour‐associated macrophages (TAMs), promote carcinogenesis by stimulating angiogenesis, extracellular matrix (ECM) remodelling, migration, invasion and metastasis 8, 9, 10, 15, 16, 17, 18. Diametrically polarized M1 macrophage and M2 macrophage are the extremes of a continuum of functional states 19. However, recent reviews on TAMs demonstrate that in marked contrast to this binary M1/M2 definition, there are some intermediate phenotypes emerging due to the plasticity of macrophages, in order to adjust to the specific microenvironment 15, 16, 17, 18. The role of these subpopulations in tumour progression remains to be carefully elucidated.

Recently, there have been extensive studies on the relevance between intratumoural macrophage infiltrations and prognosis, but the results were heterogeneous because CD68, the most extensively used macrophage marker, is expressed on all macrophages, which does not allow for the discrimination between M1 and M2 macrophage subsets 20, 21, 22, 23, 24. Tumour‐associated macrophages, on the other hand, display the alternative activated M2 macrophages in most tumours 25, 26. The M2‐polarized macrophages promote tumour progression and metastasis and can be used as prognostic indicators 27, 28, 29. M2 macrophages express markers such as mannose receptor (MR) (CD206) and haemoglobin/haptoglobin scavenger receptor (CD163) 30. But the M1‐polarized cells also participate in the process of tumour progression and have high microbicidal activity, immunostimulatory functions and tumour cytotoxicity. The classically activated M1 macrophages are characterized by CD11c and TLR4 on their surface 31, 32, 33. Based on this knowledge, we hypothesized that CD68‐positive pan‐macrophages comprise CD11c‐positive M1 macrophages and CD206‐positive M2 macrophages, and thus polarized‐TAM‐based immune status may be associated with the progression and prognosis of HCC. Our data showed that both low CD11‐positive macrophages and high CD206‐positive macrophages are associated with poor survival, whereas CD68‐positive macrophages had no prognostic significance. Furthermore, we combine with the TNM staging system and the BCLC staging system to further reflect the prognostic value of the diametrically polarized macrophages for patient outcome. We, therefore, aimed to identify a molecular biomarker able to provide a more comprehensive understanding of tumour biology and more accurate prognostic significance.

## Materials and methods

### Patients and tissue samples

We retrospectively recruited 80 consecutive patients who received curative hepatectomy between 2005 and 2009 at the Department of Hepatic surgery, Affiliated Anhui Provincial Hospital of Anhui Medical University, Hefei, China. These HCC samples were reviewed independently by two pathologists (more than 10‐year experience) without knowledge of the patients' outcome. None of these patients received any anticancer therapy before surgery. For each patient, the clinicopathological features including age, gender, tumour size, tumour differentiation, alpha‐fetoprotein, hepatitis B surface antigen, liver cirrhosis, tumour capsule, vascular invasion, Eastern Cooperative Oncology Group performance status (ECOG‐PS), BCLC classification system and TNM staging were collected retrospectively. The BCLC stage 0 is not included in this cohort because the tumours of these patients are difficult to be found or accept minimally invasive therapy (*i.e*. radiofrequency ablation and microwave coagulation). Detailed demographic and clinicopathologic information of patients is shown in Table 1. The mean age of this cohort was 57 years (range, 30–79 years), and 71.25% of the cohort was men. Patients were monitored until May 2014. Median follow‐up was 31 months (range, 1–54 months). Overall survival (OS) time was defined as the time from the date of surgery to the date of death from HCC or last visit. Recurrence‐free survival (RFS) was defined as the interval between the dates of operation and recurrence or between the dates of operation and the last observation for patients without recurrence. For each patient, informed consent was obtained before surgery. The study protocol was approved by the Human Research Ethics Committee of Anhui Medical University.

**Table 1:** Descriptive statistics of immunohistochemical variables

Variable^a^	Mean	S.D.	Median	Range
CD68	70.05	25.08	52	5–114
CD11c	23.97	9.04	21	3–55
CD206	53.45	21.73	30	1–86

a Number of cells per field (×200).

### Immunohistochemistry and evaluation

Immunohistochemical staining was used to identify and quantify infiltration of polarized TAMs in HCC. Formalin‐fixed and paraffin‐embedded samples were cut into 5‐μm sections, which then were deparaffinized in xylene and hydrated to distilled water. After the endogenous peroxidase was inhibited by 3% H_2_O_2_ for 10 min., the sections were heated in a microwave oven for 5 min. in unmasking solution (0.01 M sodium citrate buffer, pH = 6.0) and then incubated with 10% normal goat serum for 20 min. Primary monoclonal antibodies against human CD68 (KP1, 1:500; Abcam, Cambridge, MA, USA), CD11c (EP1347Y, 1:100; Abcam) and primary polyclonal antibodies against human CD206 (ab64693, 1:200; Abcam) were applied overnight at 4°C. Then, the sections were counterstained with haematoxylin, dehydrated and mounted. Negative controls were treated identically but with the primary antibody omitted.

The density of positive staining macrophages was measured using a computerized image system composed of an Olympus CCD camera DP72 and an Olympus FRAME BX53 microscope (Olympus Microsystems Imaging Solutions Ltd, Tokyo, Japan). Cells were counted under ×200 high magnifications, and to select the five independent microscopic fields with the strongest and most uniform infiltration of TAMs to ensure representativeness and homogeneity. Identical settings were used for each photograph. The density was recorded as the average count of positive cells per field. Counting of immunostained samples was performed by two pathologists (more than 10‐year experience) without the knowledge of patients' outcome and clinicopathological characteristics. For immunohistochemical density, the median value was viewed as the cut‐off for high‐/low‐expression subgroups.

### Statistical analysis

Statistical analysis was performed with SPSS 17.0 (SPSS, Chicago, IL, USA) and Prism 5 (GraphPad Software La Jolla, CA, USA). Correlations between immunohistochemical variables and clinicopathologic characteristics were analysed with the chi‐square test and Student's *t*‐tests. Continuous data were presented as mean ± S.D. We compared the immunohistochemical variables among groups by Kruskal–Wallis rank‐sum test with Bonferroni adjustment for multiple comparisons to control value or the Mann–Whitney *U*‐test, and correlations were assessed with Spearman's rank correlation for non‐normally distributed data. Kaplan–Meier analysis and log‐rank test were used to assess survival rate. Significant variables from the univariate analysis were included in the multivariate analysis when performing forward stepwise Cox regression model. All statistical analyses were two‐sided, and *P* < 0.05 was considered statistically significant. Results are reported according to Reporting Recommendations for Tumor Marker Prognostic Studies (REMARK) guidelines 34.

## Results

### Immunohistochemical characteristics

**Figure 1:**
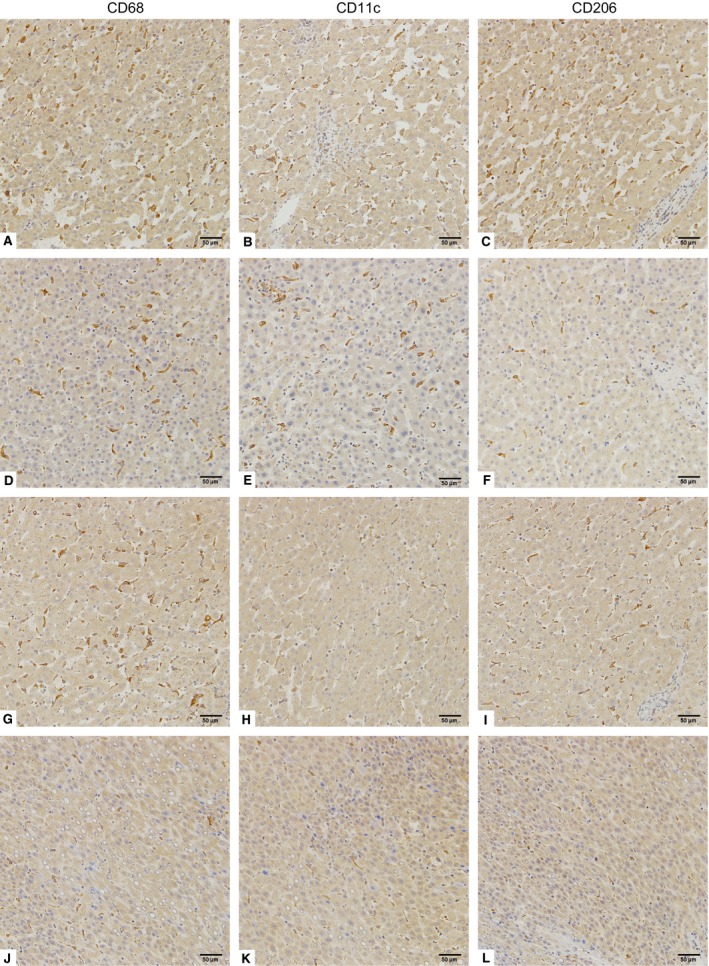
Polarized macrophage infiltration in HCC tissues. Representative images of CD68 (**A**,** D**,** G** and **J**), CD11c (**B**,** E**,** H** and **K**) and CD206 (**C**,** F**,** I** and **L**) immunohistochemical staining in HCC tissue (original magnification ×200). (**A**–**C**) show high densities of CD68‐, CD11c‐ and CD206‐positive macrophages. (**D**–**F**) show high densities of CD68‐ and CD11c‐positive macrophages, but low CD206‐positive macrophage density. (**G**–**I**) show high densities of CD68‐ and CD206‐positive macrophages, but low CD11c‐positive macrophage density. (**J**–**L**) show low densities of CD68‐, CD11c‐ and CD206‐positive macrophages.

**Figure 2:**
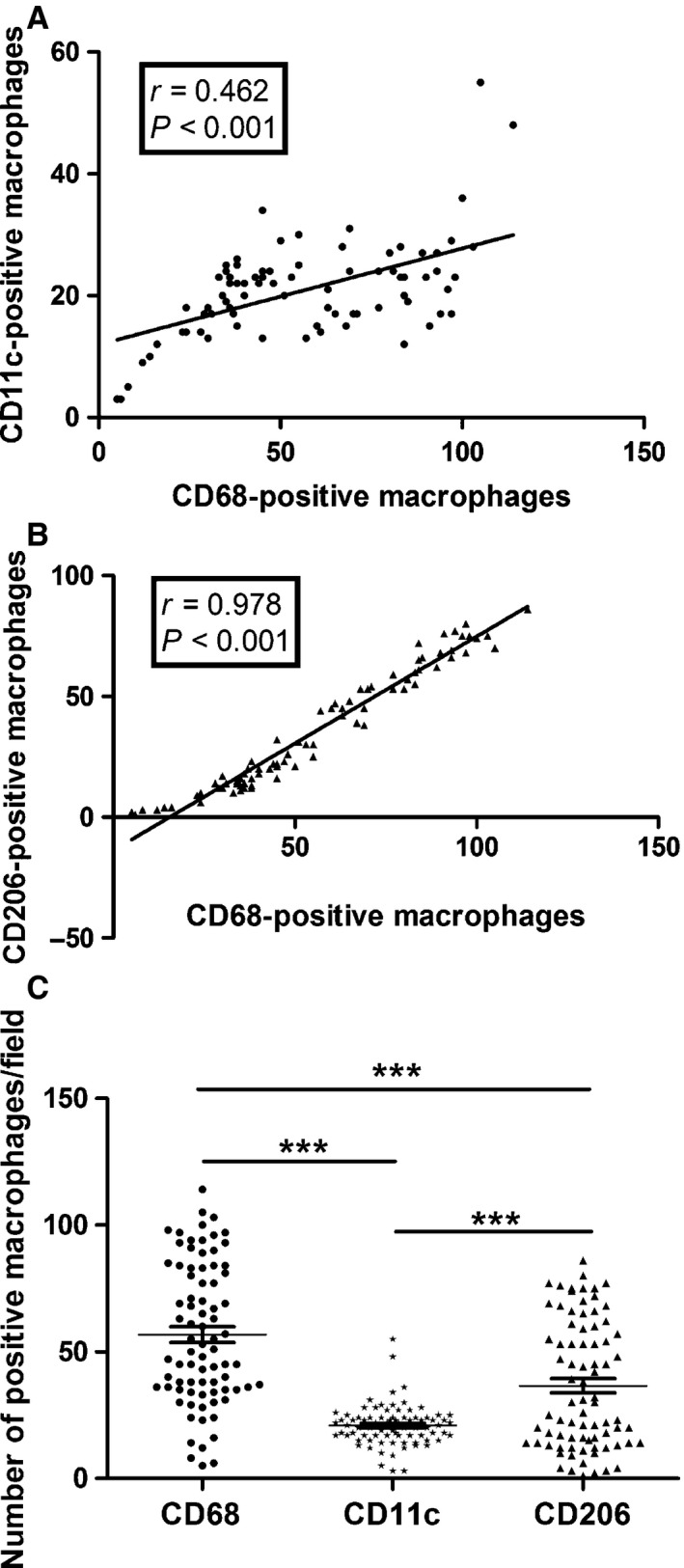
Distribution of CD68‐positive, CD11c‐positive and CD206‐positive macrophages. (**A**) Correlations between the distribution of CD68‐positive, CD11c‐positive and CD206‐positive macrophages of each case are shown. The numbers of CD11c‐positive macrophages were significantly correlated with the numbers of CD68‐positive macrophages (*r* = 0.462, *P* < 0.001). (**B**) The numbers of CD206‐positive macrophages were significantly correlated with the numbers of CD68‐positive macrophages (*r* = 0.978, *P* < 0.001). (**C**) The mean number of CD68‐positive macrophages, CD11c‐positive macrophages and CD206‐positive macrophages was 70.05 ± 25.08 (median, 52; range, 5–114), 23.97 ± 9.04 (median, 21; range, 3–55) and 53.45 ± 21.73 (median, 30; range, 1–86), respectively. ***P < 0.001.

**Figure 3:**
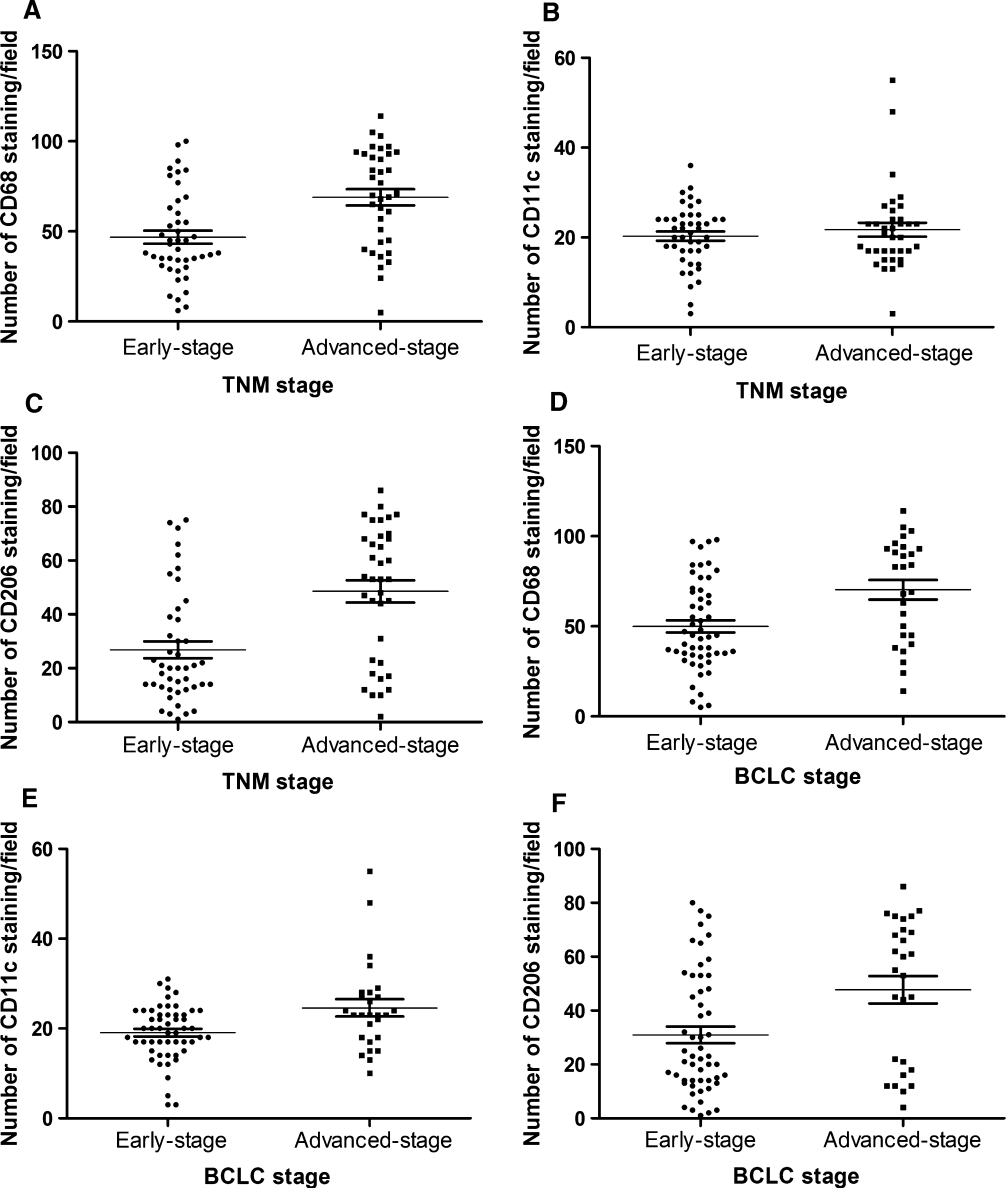
Polarized macrophage infiltration in different tumour stages. Cell counts of accumulating macrophages. Each point represents the cell count of infiltrating macrophages (/field). Bar indicates mean ± S.D. (**A** and **D**) CD68‐positive macrophages in TNM stages and BCLC stages (*P* < 0.001, *P* = 0.003, respectively); (**B** and **E**) CD11c‐positive macrophages in TNM stages and BCLC stages (*P* = 0.938, *P* = 0.015, respectively); (**C** and **F**) CD206‐positive macrophages in TNM stages and BCLC stages (*P* < 0.001, *P* = 0.011, respectively).

CD68‐, CD11c‐ and CD206‐positive macrophage staining is mainly seen in the cytoplasm and/or membrane of macrophages (Fig. 1). The distribution of CD11c‐positive macrophages, CD206‐positive macrophages and CD68‐positive macrophages in each tumour was forcefully correlated (*P* < 0.001, *r* = 0.462; *P* < 0.001, *r* = 0.978) (Fig. 2A and B). The density of CD68‐positive macrophages, CD11c‐positive macrophages and CD206‐positive macrophages was 70.05 ± 25.08 (median, 52; range, 5–114), 23.97 ± 9.04 (median, 21; range, 3–55) and 53.45 ± 21.73 (median, 30; range, 1–86), respectively (Fig. 2C, Table 1). These findings indicated that CD11c‐positive macrophages and CD206‐positive macrophages show the same localization bias as CD68‐positive macrophages in HCC tissues. Some patients are with high expression of M2 macrophages (Fig. 1G–I), some patients are with high expression of M1 macrophages (Fig. 1D–F), and some patients are with high or low expression of both M1 and M2 (Fig. 1A–C, J–L, respectively). Generally, CD68‐positive cells were more ample than CD11c‐positive or CD206‐positive cells, and CD206‐positive cells were outnumbered by CD11c‐positive cells in HCC tissues. The immunohistochemical number of CD68‐ and CD206‐positive staining exhibits significant difference among samples of different TNM stage and BCLC stage (Fig. 3). CD11c‐positive staining exhibits significant difference among samples of different BCLC stage, but it also shows no significant difference among samples of different TNM stage (Fig. 3).

### Correlations between TAM polarization status and clinicopathological features

Correlations between immunohistochemical variables and clinicopathological features were analysed with chi‐square test and are summarized in Table 2. CD68‐positive staining was positively correlated with advanced‐stage cancer (*P* = 0.002). CD11c‐positive staining was negatively correlated with the BCLC stage (*P* = 0.022). CD206‐positive staining was positively correlated with age, tumour size, vascular invasion, status of metastasis and TNM stage (*P* = 0.003, *P* = 0.036, *P* = 0.026, *P* = 0.007 and *P* < 0.001, respectively).

**Table 2:** Correlations between CD68‐, CD11c‐, and CD206‐positive macrophages expression and clinicopathological characteristics in patients with hepatocellular carcinoma after curative resection (*n* = 80)

Variables	CD68		*P*	CD11c		*P*	CD206		*P*
	Low (*n* = 40)	High (*n* = 40)		Low (*n* = 41)	High (*n* = 39)		Low (*n* = 41)	High (*n* = 39)	
Age (years)									
≤50	12	20	0.068	13	19	0.121	10	22	0.003
>50	28	20		28	20		31	17	
Gender									
Male	26	31	0.217	26	30	0.243	25	31	0.097
Female	14	9		14	9		15	8	
HBsAg									
Positive	29	31	0.606	30	30	0.698	29	31	0.366
Negative	11	9		11	9		12	8	
Cirrhosis									
Present	27	30	0.459	29	28	0.916	28	29	0.549
Absent	13	10		12	11		13	10	
AFP (ng/ml)									
≤20	21	14	0.115	21	14	0.167	20	15	0.352
>20	19	26		20	25		21	24	
Tumour size (cm)									
≤5	20	12	0.068	19	13	0.235	21	11	0.036
>5	20	28		22	26		20	28	
Tumour capsule									
Complete	28	21	0.108	26	23	0.684	28	21	0.185
Incomplete	12	19		15	16		13	18	
Vascular invasion^a^									
Present	14	19	0.216	16	17	0.678	12	21	0.026
Absent	26	21		25	22		29	18	
Edmondson grade									
I–II	28	20	0.068	28	20	0.121	27	21	0.273
III–IV	12	20		13	19		14	18	
Status of metastasis									
Present	17	24	0.117	21	20	0.996	15	26	0.007
Absent	23	16		20	19		26	13	
ECOG‐PS									
0	24	20	0.369	23	21	0.840	26	18	0.121
≥1	16	20		18	18		15	21	
TNM stage									
I–II	29	15	0.002	22	22	0.805	31	13	0.000
III–IV	11	25		19	17		10	26	
BCLC stage									
A‐B	30	23	0.098	32	21	0.022	31	22	0.069
C‐D	10	17		9	18		10	17	

a Microscopic and macroscopic tumour thrombus.

HBsAg: hepatitis B surface antigen; AFP: alpha‐fetoprotein; ECOG‐PS: Eastern Cooperative Oncology Group performance status; TNM: tumour–node–metastasis; BCLC: Barcelona Clinic Liver Cancer.

### Prognostic value of TAM polarization status and survival analysis

**Figure 4:**
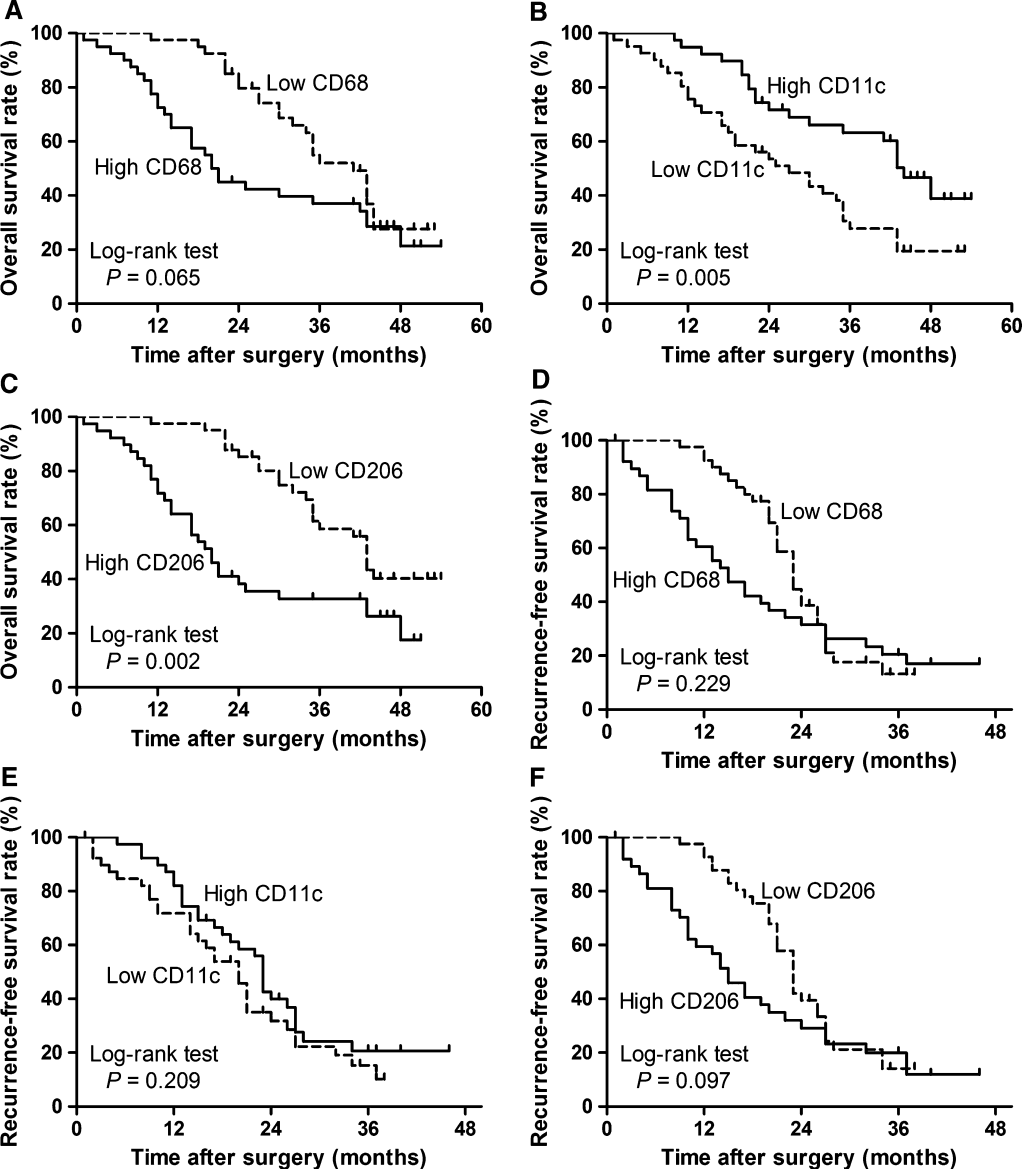
Cumulative overall survival and recurrence‐free survival curves are shown for HCC patients with diametrically polarized TAMs. Kaplan–Meier survival estimates and log‐rank tests were used to analyse the prognostic significance of CD68‐positive macrophages, CD11c‐positive macrophages and CD206‐positive macrophages. (**A** and **D**) CD68‐positive macrophages (high *versus* low, *P* = 0.065; high *versus* low, *P* = 0.229); (**B** and **E**) CD11c‐positive macrophages (high *versus* low, *P* = 0.005; high *versus* low, *P* = 0.209); (**C** and **F**) CD206‐positive macrophages (high *versus* low, *P* = 0.002; high *versus* low, *P* = 0.097). Data were dichotomized at the median value for each parameter. Dotted line, low group; solid line, high group.

**Figure 5:**
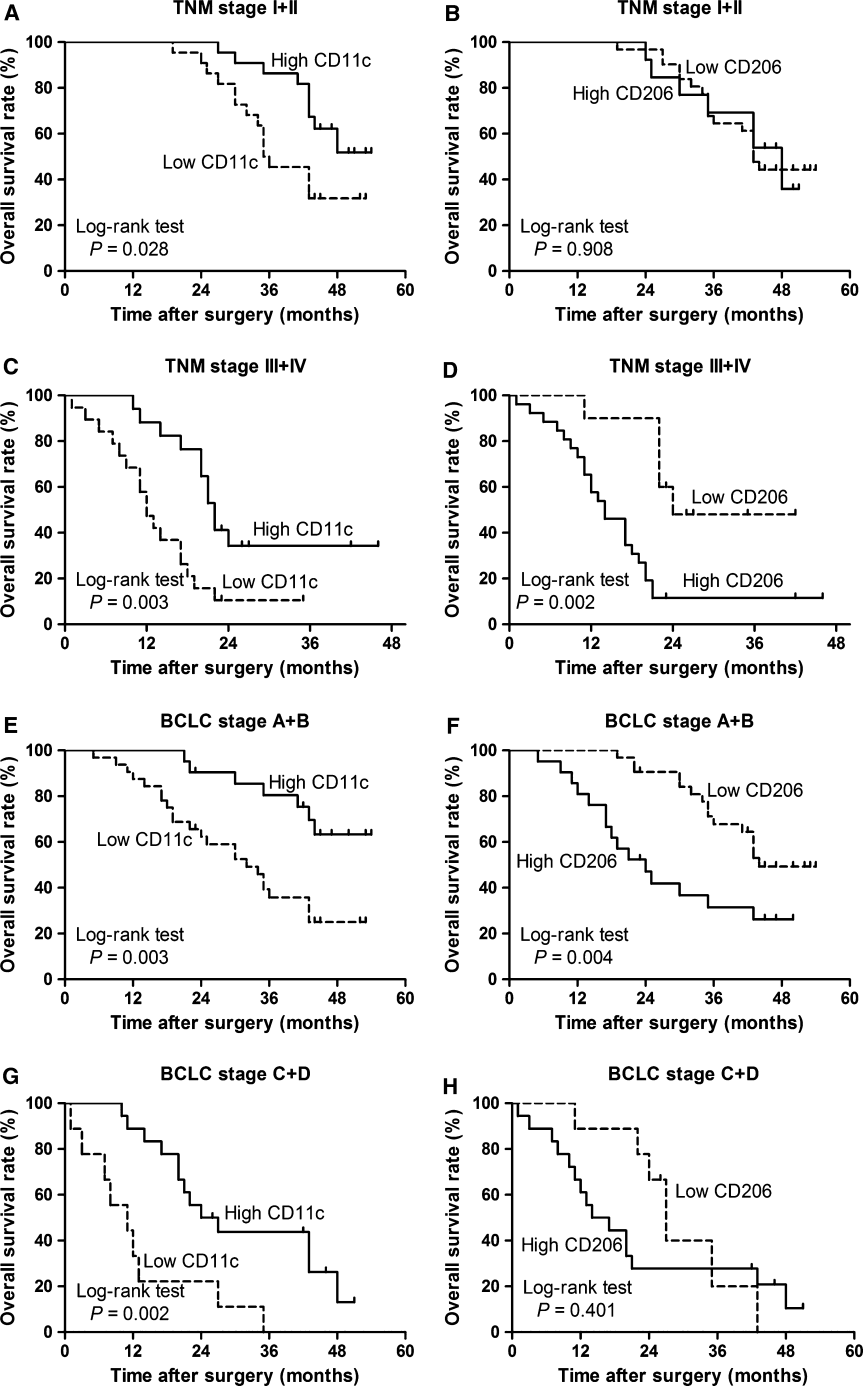
Cumulative overall survival curve is shown for HCC patients according to infiltration of polarized TAMs in different TNM stages. Kaplan–Meier survival estimates and log‐rank tests were used to analyse the prognostic significance of polarized TAMs in each subgroup. (**A** and **E**) Patients with early‐stage tumour (TNM stages I and II; BCLC stages A and B) according to CD11c expression (high *versus* low, *P* = 0.028; high *versus* low, *P* = 0.003); (**B** and **F**) patients with early‐stage tumour (TNM stages I and II; BCLC stages A and B) according to CD206 expression (high *versus* low, *P* = 0.908; high *versus* low, *P* = 0.004); (**C** and **G**) patients with advanced‐stage tumour (TNM stages III and IV; BCLC stages C and D) according to CD11c expression (high *versus* low, *P* = 0.003; high *versus* low, *P* = 0.002); (**D** and **H**) patients with advanced‐stage tumour (TNM stages III and IV; BCLC stages C and D) according to CD206 expression (high *versus* low, *P* = 0.002; high *versus* low, *P* = 0.401). Dotted line, low group; solid line, high group.

To investigate the association of diverse TAM polarization status with HCC progression, we divided 80 HCC patients into two groups based on the median values of CD68‐positive macrophages, CD11c‐positive macrophages and CD206‐positive macrophages, respectively. Kaplan–Meier survival curves were then plotted to further investigate associations with OS and RFS (Fig. 4). The log‐rank test was used to compare survival curves. Low CD11c‐positive macrophage density or high CD206‐positive macrophage density was associated with reduced survival rate (Fig. 4B and C; *P* = 0.005 and *P* = 0.002, respectively), whereas CD68‐positive staining has no significant relation with OS (Fig. 4A; *P* = 0.065). Moreover, the presence of these positive staining macrophages did not show any prognostic significance for RFS (all *P* > 0.05, Fig. 4D–F). In order to investigate further the effect of diametrically polarized TAMs in stratifying patients with different TNM stages and BCLC stages, we considered that the early‐stage tumour includes TNM stages I/II and BCLC stages A/B and advanced‐stage tumour includes TNM stages III/IV and BCLC stages C/D. In TNM stages, CD11c‐positive staining macrophages were positively correlated with OS and CD206‐positive staining macrophages had no significant relation with OS in patients with early‐stage tumour (Fig. 5A and B; *P* = 0.028 and *P* = 0.908, respectively). Moreover, CD11c‐positive staining macrophages were positively correlated with OS and CD206‐positive staining macrophages were negatively correlated with OS in patients with advanced‐stage tumour (Fig. 5C and D; *P* = 0.003 and *P* = 0.002, respectively). In BCLC stages, CD11c‐positive macrophages were positively correlated with OS in patients with early‐stage and advanced‐stage tumour (Fig. 5E and G; *P* = 0.003 and *P* = 0.002, respectively). However, CD206‐positive macrophages were negatively correlated with OS in patients with early‐stage tumour, and it had no significant relation with OS in patients with advanced‐stage tumour (Fig. 5F and H; *P* = 0.004 and *P* = 0.401, respectively). These applications demonstrate that diversely polarized TAMs might provide additional prognostic information in different tumour stages.

Univariate analysis of prognostic factors indicated that vascular invasion (*P* < 0.001), status of metastasis (*P* < 0.001), TNM stage (*P* < 0.001), BCLC stage (*P* = 0.002), ECOG‐PS (*P* = 0.008), CD11c‐positive expression (*P* = 0.006) and CD206‐positive expression (*P* = 0.003) had significant prognostic influence on OS (Table 3). Moreover, multivariate survival analysis revealed that only TNM stage (*P* < 0.001), BCLC stage (*P* = 0.007), CD11c‐positive expression (*P* < 0.001) and CD206‐positive (*P* = 0.031) expression were identified as independent predictors of poor prognosis for OS in HCC patients after adjustment of covariates (Table 3).

**Table 3:** Univariate and multivariate Cox proportional hazard regression analysis of patients' overall survival

Variables	Univariate			Multivariate		
	HR	95% CI	*P*‐value	HR	95% CI	*P*‐value
Age (years) (≤50 *versus* >50)	1.087	0.635–1.861	0.761			
Gender (female *versus* male)	1.204	0.691–2.098	0.512			
HBsAg (negative *versus* positive)	0.991	0.541–1.816	0.977			
Cirrhosis (absent *versus* present)	1.155	0.658–2.028	0.615			
AFP (ng/ml) (≤20 *versus* >20)	0.905	0.534–1.534	0.710			
Tumour size (cm) (≤5 *versus* >5)	1.246	0.733–2.116	0.416			
Tumour capsule (incomplete *versus* complete)	1.178	0.684–2.029	0.554			
Vascular invasion (absent *versus* present)	0.341	0.196–0.592	<0.001	0.780	0.290–2.102	0.624
Edmondson grade (I/II *versus* III/IV)	1.020	0.594–1.754	0.942			
Status of metastasis (absent *versus* present)	0.298	0.171–0.522	<0.001	1.808	0.589–5.548	0.300
ECOG‐PS (≤0 *versus* >1)	0.489	0.288–0.830	0.008	0.652	0.261–1.630	0.361
TNM stage (I/II *versus* III/IV)	0.180	0.099–0.326	<0.001	5.173	2.661–10.059	<0.001
BCLC stage (A/B *versus* C/D)	0.426	0.248–0.730	0.002	2.625	1.294–5.324	0.007
CD68‐positive macrophages (low *versus* high)	0.636	0.376–1.077	0.092			
CD11c‐positive macrophages (low *versus* high)	2.128	1.241–3.649	0.006	0.178	0.086–0.367	<0.001
CD206‐positive macrophages (low *versus* high)	0.447	0.263–0.760	0.003	1.943	1.062–3.552	0.031

CI: confidence interval; HR: hazard's ratio; HBsAg: hepatitis B surface antigen; AFP: alpha‐fetoprotein; ECOG‐PS: Eastern Cooperative Oncology Group performance status; TNM: tumour–node–metastasis; BCLC: Barcelona Clinic Liver Cancer.

## Discussion

In this study, we found that the infiltration of polarized TAMs influences OS in patients with HCC. Moreover, we observed that CD68‐positive macrophages have no significant correlation with OS, whereas CD11c‐positive macrophages or CD206‐positive macrophages have a significant positive and negative correlation with OS, respectively. Cox proportional hazards regression analysis confirmed that the polarized TAMs emerged as an independent prognostic factor. Combining the polarized TAMs with the TNM stage and the BCLC stage can help us to further quantify the prognostic risk and provide more prognostic information. However, its value requires independent and more data to validate it.

Depending on different external stimuli milieu, macrophages can acquire different phenotypes and possess opposing immune function properties 35. M1 macrophages produce pro‐inflammatory cytokines, exhibit strong microbicidal properties and mediate resistance to pathogens and tumour cytotoxicity. M1‐polarized macrophages occur when the cells receive stimuli such as (*i*) IFN‐γ, mainly secreted by T‐helper (Th)1 cells, cytotoxic T cells and natural killer cells; (*ii*) LPS, which constitute a significant component of the outer membrane of Gram‐negative bacteria; and (*iii*) granulocyte‐macrophage CSF that stimulates the production of many kinds of pro‐inflammatory cytokines 36, 37, 38. M1 macrophages secrete cytokines such as IL‐1β, TNF, IL‐6, leukotriene B4 (LTB4) and nitric oxide; they express high levels of CD11c in addition to CD11b and F4/80 39. The M2 macrophage activation is induced by fungi, parasites, immune complexes, apoptotic cells, M‐CSF‐1, IL‐4, IL‐13, IL‐10, tumour growth factor beta and glucocorticoid 40; M2 macrophages can express high levels of IL‐10, IL‐1 receptor antagonist, MRs (CD206), arginase‐1 and CD163 antigen 41. The M2 macrophages have phagocytosis capacity, producing ECM components, angiogenic and chemotactic factors, and IL‐10 42. M2 macrophages can mitigate inflammatory response, clear apoptotic cells and promote wound healing 35, 43. In the current literature, they are widely termed as anti‐inflammatory, wound healing and tissue repair; promote tumour progression and metastasis; and are considered as benign opposites of the M1‐activated macrophages 31, 44. In accordance with this notion, our data showed that CD206‐positive macrophages were more abundant than CD11c‐positive macrophages in most cases, indicating an M2‐polarized macrophage infiltration in HCC tissues. Conversely, CD11c‐positive macrophages overnumbering CD206‐positive macrophages were also observed in a few cases, suggesting the existence of TAMs with relatively M1‐skewed phenotype macrophages. These data illustrate that single use of a marker to evaluate the density of macrophages may not reflect actual macrophage situation in the tumour microenvironment. Moreover, prognostic significance of TAMs in human cancers has been recently critically evaluated 45. Accumulating epidemiological evidence indicated that high numbers of TAMs are significantly associated with poor patient prognosis in human cancers, such as breast, gastric and bladder 16, 46. In contrast, other studies have revealed that the prognostic significance of TAMs can be controversial 45. For example, in patients with high‐grade osteosarcoma, CD68‐positive macrophages have been statistically significantly correlated with better survival, whereas the number of CD68‐positive macrophages has been positively correlated with clinical outcome in patients with large B‐cell lymphoma 47, 48. In gastric cancer, a study revealed that the number of TAMs was found to be independent predictor of patient better survival 49, and another study found a negative correlation between TAMs and prognosis 50. In colorectal cancer, the prognostic significance of TAMs was controversial and found that TAMs could depend on distinct phenotypes acquired on distinct microlocalization within the tumour 51. However, the diametrically polarized TAMs with opposed functional status were beyond these studies. Recently, some studies reported the prognostic significance of M1/M2 phenotypes using combined analysis of CD11c and CD206 in renal cell carcinoma and gastric cancer 52, 53. This may be better to help us clarify the prognostic significance of diametrically polarized macrophages. In this study, we found that diametrically polarized macrophages (M1/M2 phenotypes) can be incorporated with TNM stage and BCLC stage to further quantify the prognostic risk for OS.

In this study, we found that the generic macrophage marker CD68 expression only correlated with TNM stage in clinicopathological characteristics. In contrast, low CD11c expression correlated with BCLC stage, and high CD206 expression correlated with age, tumour size, vascular invasion, status of metastasis, TNM stage and BCLC stage (Table 1). These results partially revealed the correlation between the effects of polarized TAMs and the tumour cell biological phenotype. Because CD68 marker does not distinguish between M1 and M2 subpopulation and not all TAMs exhibit M2 phenotype, some TAMs show their M1 phenotype tumouricidal behaviour and inhibit tumour growth, and they can also be stained by CD68. The absence of a specific marker for M1‐polarized macrophages is a difficult problem in macrophage research.

However, CD11c is a member of the β2 integrins and commonly used as a marker for M1 macrophages 54, 55, 56, 57. As M1 macrophages are accredited to a profound regulatory effect on T cell tumouricidal responses, one might expect an increased M1 infiltration in early‐stage cases. Our results showed a significantly higher CD11c expression in the early‐stage HCC with BCLC stages and TNM stages. When describing an increased macrophage infiltration and a shift towards the M2‐polarized phenotype in advanced‐stage cancers, the increased CD11c macrophage also needs to be discussed. A study also report CD11c as marker for dendritic cells 58, so one fraction of CD11c marker might represent dendritic cell subpopulations. One group analysed the gene expression profile of CD11c low and CD11c high macrophages in decidual tissue 59, and they detected differential gene expression profiles in both subtypes. Interestingly, both cell types expressed pro‐ and anti‐inflammatory cytokines. The authors deduce that they do not fit into the classical M1–M2 allocation 59. However, one group used CD11c as an M1 marker in flow cytometric analysis and reported that diet‐induced obesity results in a shift in the state of adipose tissue macrophages from an M2‐polarized state to an M1‐polarized state that contributes to insulin resistance 39. Based on the available data and some recent reports, we can conclude that CD11c may be considered a marker for M1‐polarized macrophages, but whether it is one of the best reliable indicators still needs further research.

CD206 (also known as macrophage MR) belongs to the MR family and is a 175‐kD type I transmembrane receptor with three types of extracellular domains: a functional cysteine‐rich domain, eight C‐type lectin‐like domains and a fibronectin domain 60, 61, and commonly used as a marker for M2 macrophages 52, 53, 62, 63. Some studies have also reported that M2 macrophages correlated negatively with the patient prognosis 52, 53, 64. Moreover, another group reported that macrophages in human subcutaneous fat tissue that accumulated with fat mass development exhibit a particular M2 phenotype, with CD206 as a M2‐macrophage marker 65. Consistent with these observations, our study showed that CD206 expression positively correlated with age, tumour size, vascular invasion, status of metastasis, TNM stage and BCLC stage. Moreover, we observed that CD206‐positive macrophages have a significant negative correlation with OS in HCC. Our findings indicated a potent pro‐tumoural macrophage phenotype marker, CD206, and a potent anti‐tumoural macrophage phenotype marker, CD11c.

Accumulating evidence indicates that the success of anticancer therapies, including radiotherapy, cytotoxic compounds and targeted agents, may depend on the activation of anticancer immune responses 66. However, the phenotype of M1‐polarized or (and) M2‐polarized macrophages can, to some extent, be reversed *in vivo* and *in vitro*. Thus, restoration of the complex immune system to the anti‐tumour state by polarized macrophage may open a new avenue for treatment of patients with HCC.

### Limitations of the study

The total number of patients (80 tumour resection specimens) included in this retrospective study was relatively small, and patient selection bias is likely present. This study is based on one methodology: counting of immunohistochemically stained macrophages in HCC tissue. A large patient selection and many methodologies could eventually reveal such results.

## Conclusion

Our study shows the prognostic significance of polarized TAMs in HCC. Tipping TAMs towards an anti‐tumoural phenotype might be a feasibility of a possible new immunotherapeutic target for postoperative treatment. Combining the polarized TAMs with the proven TNM stage and BCLC stage can help us to further quantify the prognostic risk and provide more prognostic information, intervention counselling for patients, selecting patients for adjuvant therapies, and customizing follow‐up after surgery.

## Conflicts of interest

The authors declare no conflicts of interest.
